# Comparative Effects of Core Versus Forearm Training on Pull-Up Repetition Performance in Physically Inactive Males

**DOI:** 10.3390/sports13120433

**Published:** 2025-12-04

**Authors:** Hamidreza Sepehri Rahnama, Sayyedarmin Ganji, Kitty Vadasz, Judit Prokai

**Affiliations:** Institute of Sport Sciences and Physical Education, Faculty of Sciences, University of Pécs, 7624 Pécs, Hungary; arminganji01@gmail.com (S.G.); vadaszki@gamma.ttk.pte.hu (K.V.); prokai@gamma.ttk.pte.hu (J.P.)

**Keywords:** pull-up, grip strength, forearm training, core stability, isometric endurance, upper-body performance, resistance training

## Abstract

Pull-ups are a widely recognized exercise for training and assessing upper-body strength and muscular endurance, requiring coordinated activation of the latissimus dorsi, biceps brachii, forearm flexors, and core stabilizers. However, many individuals experience difficulty performing pull-ups due to inadequate grip strength or core stability. This study aimed to investigate the comparative effects of forearm-specific and core-specific training, combined with standardized pull-up routines, on upper-body performance indicators in physically inactive male students. Thirty participants (age = 21 ± 1.58 years) were randomly assigned to three groups (*n* = 10): pull-up plus interval training (PIT), pull-up plus forearm training (PFT), and pull-up plus core training (PCT). All groups performed identical pull-up-based programs for eight weeks, differing only in the supplemental exercises. Before and after the intervention, participants were tested for maximum pull-up repetitions, grip strength (both hands), and dead-hanging time. Significant improvements were found in all variables (*p* ≤ 0.009), with group differences in pull-up repetitions (*p* < 0.001) and right-hand grip strength (*p* = 0.004). The PFT group achieved the greatest gains, with a 222.5% increase in repetitions, 12% and 14.0% increases in right- and left-hand grip strength, and a 55.3% increase in hanging time. The PCT group showed moderate progress, while PIT yielded the smallest improvement. Forearm-specific training proved the most effective strategy for enhancing pull-up performance, grip strength, and endurance.

## 1. Introduction

Pull-ups (PU) are a fundamental exercise for training and assessing upper-body strength and muscular endurance [1]. They are widely employed in athletic training, military evaluations, and general fitness programs [2]. The movement primarily activates the latissimus dorsi, biceps brachii, and forearm flexors, while also requiring substantial core engagement to maintain alignment and minimize energy leakage during execution [3,4].

The importance of PU performance extends beyond general fitness. In sports such as gymnastics, swimming, climbing, and football, enhanced PU ability has been associated with improved sports performance. Greater PU strength correlates with faster sprint swimming times and improved climbing performance [5,6,7]. Additionally, strong upper-body pulling strength is linked to increased throwing distances in football, emphasizing its sport-specific utility [8].

Despite this relevance, many individuals struggle to perform PUs due to two commonly undertrained factors: grip strength and core stability. Grip strength directly influences the ability to maintain contact with the bar, thereby affecting endurance and repetition capacity [9,10]. At the same time, core stability enhances kinetic efficiency by improving postural control and minimizing unnecessary movement [11,12,13].

Recent research has highlighted the importance of grip endurance in sustaining upper-body pulling performance [14,15]. In addition, contemporary evidence emphasizes the critical role of trunk stability in facilitating efficient force transmission during demanding upper-body tasks [11,12,13]. Together, these findings suggest that grip-specific and core-focused interventions may influence PU performance through distinct but complementary neuromuscular pathways [16].

Although previous studies have examined the independent roles of grip and core strength in PU performance, few have directly compared the effects of targeted training interventions on these factors [11,14,15,16]. Moreover, limited research has applied progressive overload principles to assess long-term training adaptations [17,18].

To address the gap in the current knowledge base, the present study examined the comparative effects of two distinct 8-week programs, one focusing on the forearm and the other on core-focused exercise protocols, each integrated with a standardized PU training regimen. The interventions were evaluated based on their impact on PU repetition count, grip strength of both hands, and hanging endurance in physically inactive male university students.

It was hypothesized that both training protocols would elicit improvements in PU performance; however, the forearm-specific intervention was expected to produce superior gains, attributable to its targeted enhancement of grip endurance, a key determinant in PU execution [16,19].

## 2. Materials and Methods

### 2.1. Participants

Thirty physically inactive male university students (defined as having no structured exercise routine and engaging only in occasional recreational activity) (age = 21 ± 1.58 years; body mass = 73.25 ± 18.03 kg; height = 176.5 ± 21.92 cm) participated in this study. Inclusion criteria included no engagement in physical training within the past six months, absence of musculoskeletal injury or chronic illness, and no history of smoking or drug use. All participants were required to adhere to the provided dietary and sleep guidelines and to refrain from any additional physical activity during the intervention period. Training attendance and exercise execution were fully supervised by both investigators at every session. Dietary and sleep adherence were monitored weekly through participant self-report logs and verbal confirmation. Written informed consent was obtained from all participants before commencing the research. The study was conducted in accordance with the Declaration of Helsinki [20]. Approval for the publication of this article was provided by the Regional and Institutional Research Ethics Committee of the Medical School, Clinical Center, University of Pécs, at the meeting held on the 12th of September 2025, as the data connected to this article are processed ethically and anonymously for scientific purposes (10211-PTE2025).

### 2.2. Study Design

This eight-week experimental study employed a stratified randomization procedure. All participants first completed a baseline maximum repetition PU test. Based on these initial results, participants were ranked and then evenly distributed into three groups (n = 10 each): Pull-up and Interval Training (PIT), Pull-up and Forearm Training (PFT), Pull-up and Core Training (PCT), to ensure comparable baseline performance across groups. Stratification was primarily determined by baseline pull-up performance to ensure balanced initial strength levels across groups. In addition, age, height, and body mass were considered during allocation to maintain comparable anthropometric characteristics among the three training groups.

The PIT group was included to equalize total training time across all participants without introducing additional resistance or muscle-specific exercises. This design ensured that all groups trained for an equal duration while avoiding potential interference that could arise if the control group performed resistance training for other muscle groups [21]. Interval training was chosen instead of a passive control to ensure that all participants completed an equivalent total training time and volume while avoiding any resistance-based or muscle-specific adaptations that could influence PU performance. Each group completed two training sessions per week (1 h per session), combining a standardized PU routine with group-specific exercises. Performance variables were assessed at baseline (week 1) and post-intervention (week 8), consisting of maximum PU repetitions, grip strength (right and left hand), and dead hanging time. To minimize fatigue-related bias, assessments were conducted on separate days under standardized environmental conditions. All tests were completed within a repeated measures design and were administered by the same assessor to ensure consistency, with a second investigator supervising all sessions. Both assessors received standardized laboratory training in test administration prior to data collection.

### 2.3. Test Procedures

#### 2.3.1. Pull-Up Repetitions

Upper-body muscular endurance was assessed using a standardized PU test [7]. Participants grasped a horizontal bar with a closed, pronated, shoulder-width grip in hanging as a starting position and performed repetitions until failure. A valid repetition required the chin to pass above the bar and the arms to fully extend during the lowering phase. Each participant completed up to three trials, with two-minute rest intervals between attempts, to ensure adequate recovery and performance consistency in line with previous resistance training research [22]. The highest repetition count was recorded.

#### 2.3.2. Grip Strength

Isometric grip strength was measured using a digital grip strength dynamometer (Model T.K.K. 5401). Participants stood upright with the elbow flexed at 90° and were instructed to squeeze maximally for three seconds. Three trials were performed per hand, with one-minute rest intervals. The highest value was recorded separately for the right and left hands, according to the standard ASHT guidelines [10].

#### 2.3.3. Dead-Hanging Time

Grip endurance was evaluated by recording the maximum duration participants could hang from a pull-up bar using a standard closed pronated grip, with the thumb wrapped around the bar to ensure safety and standardization [15]. Participants stepped onto a stable box positioned beneath the bar, were instructed to fully extend their elbows before the start, and the test began following a standardized verbal countdown (“3–2–1–hang”). Timing started once both feet left the box and continued until grip failure or voluntary release, with total duration measured in seconds.

For each test, only the best performance from the completed attempts was recorded. As a result, individual trial values were not retained, and coefficients of variation (CV) and typical error could not be calculated.

### 2.4. Training Intervention

All groups followed a standardized PU training protocol twice weekly for eight weeks [18]. Each session lasted 60 min, and combined general PU exercises with group-specific training. A two-session weekly frequency was chosen because all participants were physically inactive and could not tolerate a higher weekly load. Additionally, the volitional-failure protocol required 48–72 h of recovery between sessions, making two sessions per week the most feasible and safe option. The PU routine included assisted, inverted rows, reverse-grip, and negative PUs, adjusted to reach muscular failure within each set. Assisted pull-ups involved minimal support to complete full-range repetitions; inverted rows were performed as horizontal body pulls with feet on the ground; reverse-grip pull-ups used a supinated grip (palms facing the lifter); and negative pull-ups began from the top position using a box, with participants lowering themselves under controlled eccentric tension for several seconds before stepping back onto the box. These general PU exercises were all performed in three sets per session, with participants completing as many repetitions as possible (typically 10–12) in each set and resting 1–2 min between sets. The core exercises included V-ups (lifting the trunk and extended legs simultaneously from a supine position), leg raises (raising straight legs to roughly 90° while keeping the lower back stable), cat–cow (slow alternating spinal flexion and extension in a quadruped stance), and the forearm plank (isometric hold with elbows under shoulders and a neutral spine). Group-specific protocols are highlighted in Table 1. They include details such as “3 × 12,” which indicates 3 sets × 12 repetitions performed to volitional failure, with 1–2 min of rest between sets and 2–3 min between exercises. All resistance-based exercises (wrist curls, reverse wrist curls, barbell grip curls, and the farmer walk) used individually adjusted loads so that participants reached volitional failure within the target repetition or time range, with loads progressively increased as strength improved. High-intensity running was performed at approximately 80–90% of each participant’s estimated maximal running speed.

All protocols incorporated progressive overload principles to promote adaptation and prevent stagnation, in line with current recommendations [17].

### 2.5. Statistical Analysis

All statistical analyses were performed using OriginLab 2018 (OriginLab Corporation, Northampton, MA, USA). Shapiro–Wilk tests were used to assess normality for all variables, with PIT PU data identified as non-parametric. ANOVA assumptions, including homogeneity of variances, were verified and met before analysis. Paired *t*-tests (with Bonferroni correction) and Wilcoxon tests were applied for within-group comparisons. Between-group and time effects were analyzed using a two-way ANOVA. Statistical significance was set at *p* ≤ 0.05. Effect sizes were calculated as follows: Cohen’s dz was used for paired pre–post comparisons, r (Z/√N) for Wilcoxon tests, and eta squared (η^2^) for ANOVA. Effect sizes (Cohen’s d) were interpreted as small (≥0.20), medium (≥0.50), and large (≥0.80). Power analysis was performed for all primary outcomes, although no a priori power analysis was conducted due to the exploratory nature of the study [23].

## 3. Results

All thirty participants completed the study. No significant differences were observed in baseline anthropometric characteristics or pre-test values among the groups (Table 2).

### 3.1. PU Repetitions

**Figure 1:**
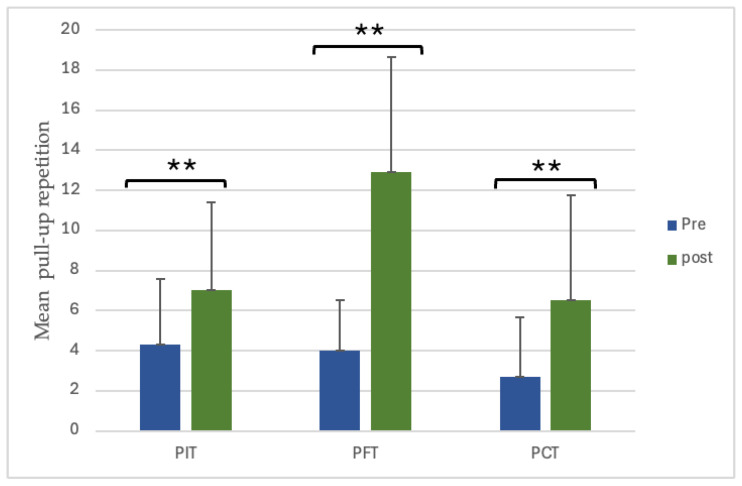
Pre- and post-training PU repetitions within each group. ** *p* < 0.01. (PU = pull-up repetitions; PIT = pull-up + interval training; PFT = pull-up + forearm training; PCT = pull-up + core training).

All groups demonstrated significant within-group improvements in PU repetitions following the 8-week intervention. PIT increased from 4.30 ± 3.27 to 7.00 ± 4.42, representing a 62.8% enhancement (*p* = 0.0069, dz = 2.02, large); PFT improved from 4.00 ± 2.54 to 12.90 ± 5.76, corresponding to a 222.5% gain (*p* < 0.001, dz = 2.39, large); and PCT improved from 2.70 ± 2.95 to 6.50 ± 5.23, an increase of 140.7% (*p* = 0.001, dz = 1.48, large) (Figure 1).

**Figure 2:**
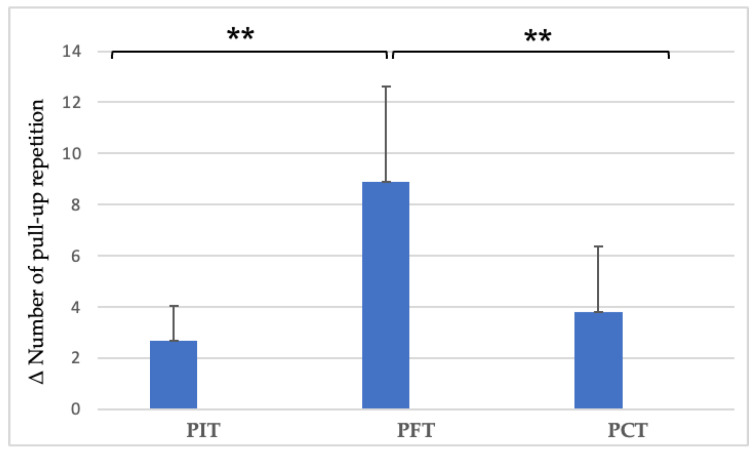
Between-group differences in PU repetition improvements after the 8-week intervention. ** *p* < 0.01. (PU = pull-up repetitions; PIT = pull-up + interval training; PFT = pull-up + forearm training; PCT = pull-up + core training; Δ = pre–post change).

Two-way ANOVA revealed a significant interaction between group and time in PU repetition improvements (F(2,27) = 14.73, *p* < 0.001). PFT exhibited the greatest enhancement, followed by PCT, whereas PIT showed the smallest improvement. Post hoc analysis indicated that the PFT group achieved significantly greater gains compared to both PIT (*p* < 0.001) and PCT (*p* < 0.001), whereas the difference between PCT and PIT was not significant (*p* = 0.73) (Figure 2).

### 3.2. Grip Strength

**Figure 3:**
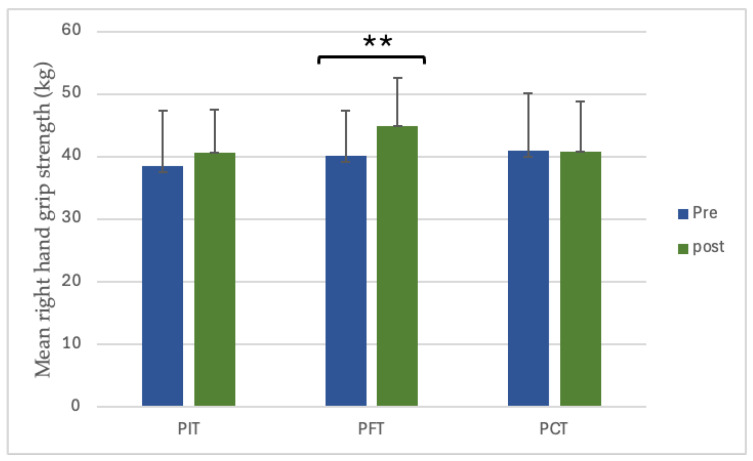
The value of pre- and post-training right-hand grip strength. ** *p* < 0.01. (PIT = pull-up + interval training; PFT = pull-up + forearm training; PCT = pull-up + core training; kg = kilograms).

**Figure 4:**
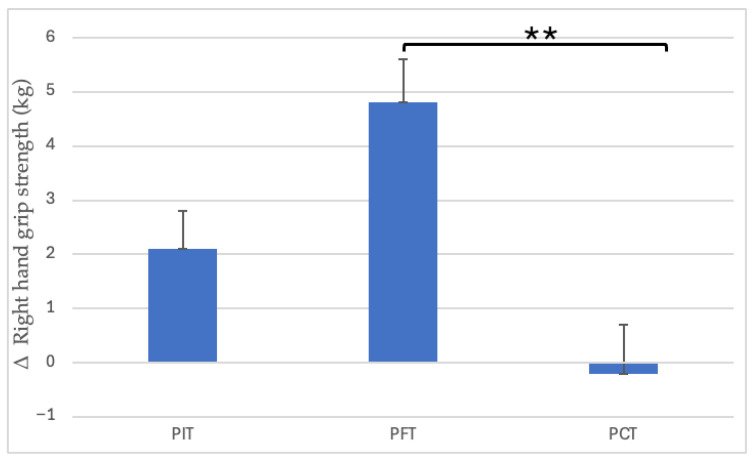
Between-group differences in right-hand grip strength improvements. ** *p* < 0.01. (PIT = pull-up + interval training; PFT = pull-up + forearm training; PCT = pull-up + core training; kg = kilograms; Δ = pre–post change).

Only the right hand in the PFT group presented significant improvement from 40.1 ± 7.23 to 44.9 ± 7.75 (12.0%, *p* < 0.001, dz = 1.68, large). PIT and PCT showed no significant change (Figure 3). There was significant interaction between group and time (*p* = 0.004), and post hoc analysis indicated that PFT significantly differed from PCT (*p* = 0.0048) (Figure 4).

**Figure 5:**
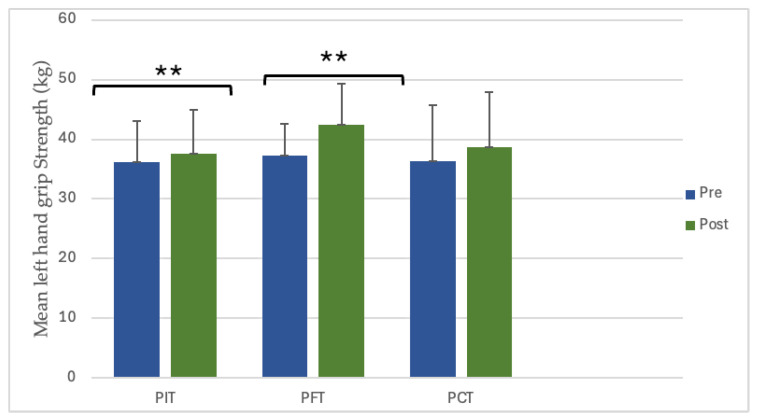
The value of pre- and post-training left-hand grip strength. ** *p* < 0.01. (PIT = pull-up + interval training; PFT = pull-up + forearm training; PCT = pull-up + core training; kg = kilograms).

**Figure 6:**
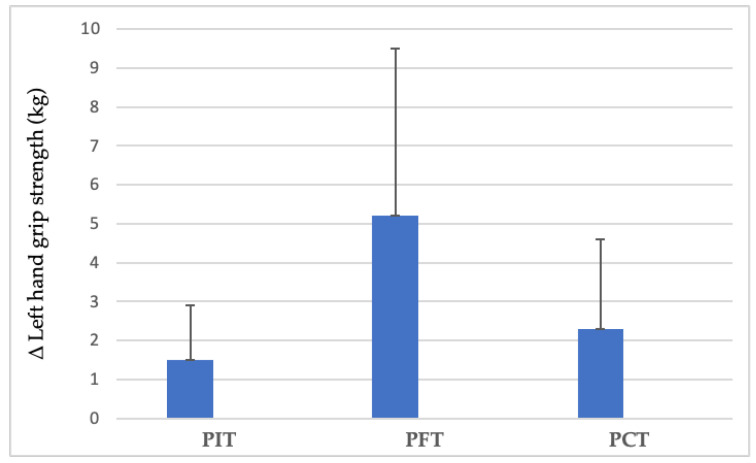
Between-group differences in left-hand grip strength improvements. (PIT = pull-up + interval training; PFT = pull-up + forearm training; PCT = pull-up + core training; kg = kilograms; Δ = pre–post change).

Left-hand grip strength demonstrated significant increases in two groups. PIT increased from 36.1 ± 6.9 kg to 37.6 ± 7.3 kg (4.2%, *p* = 0.009, dz = 1.05, large), and PFT increased from 37.3 ± 5.3 kg to 42.5 ± 6.9 kg (14.0%, *p* = 0.004, dz = 1.20, large), while PCT confirmed a non-significant improvement (Figure 5). No significant interaction between group and time was observed for left-hand gains (*p* = 0.062) (Figure 6).

### 3.3. Dead-Hanging Time

**Figure 7:**
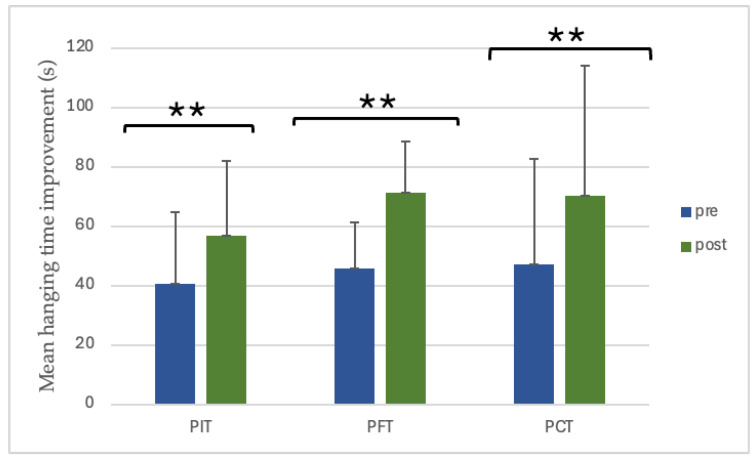
The value of pre- and post-training dead-hanging time. ** *p* < 0.01. (PIT = pull-up + interval training; PFT = pull-up + forearm training; PCT = pull-up + core training; s = seconds).

**Figure 8:**
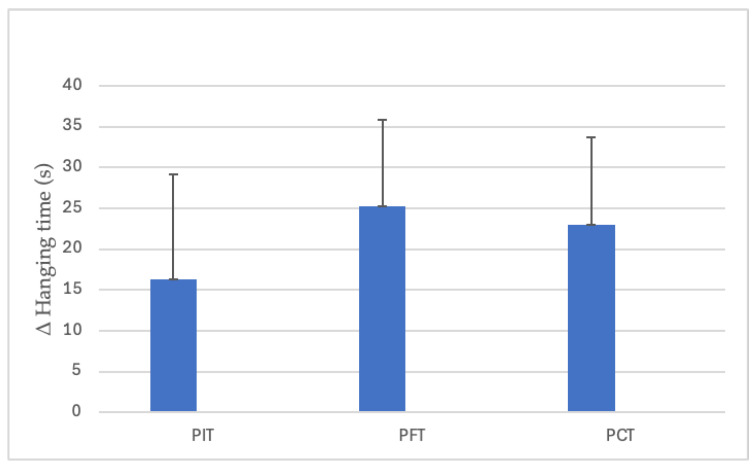
Between-group differences in dead-hanging time improvements. (PIT = pull-up + interval training; PFT = pull-up + forearm training; PCT = pull-up + core training; s = seconds; Δ = pre–post change).

The dead-hanging time significantly improved in all groups following the eight-week intervention. PIT increased from 40.4 ± 24.4 s to 56.8 ± 25.3 s (40.7%, *p* = 0.003, dz = 1.27, large), PFT from 45.8 ± 15.4 s to 71.1 ± 17.4 s (55.3%, *p* < 0.001, dz = 2.41, large), and PCT from 47.2 ± 35.4 s to 70.2 ± 43.7 s (48.7%, *p* < 0.001, dz = 2.15, large) (Figure 7). However, the interaction between group and time was observed as non-significant (*p* = 0.215) (Figure 8).

### 3.4. Statistical Power

Statistical power was high for pull-up repetitions (0.999) and right-hand grip strength (0.93), moderate for left-hand grip strength (0.59), and low for dead-hanging time (0.34).

## 4. Discussion

The principal findings of this investigation were that eight weeks of combined PU training with interval-, forearm-, or core-specific exercises resulted in significant improvements in PU repetitions, grip strength, and grip endurance. PU performance increased in all groups, with the greatest gains observed in the forearm training group; maximal grip strength improved in the forearm group (right and left) and in the interval group (left only); and hanging time improved significantly across all groups.

Participants’ body mass and height remained unchanged throughout the intervention. Consequently, changes in performance were unlikely to have been influenced by alterations in a participant’s anthropometry.

PU repetitions increased substantially in all groups, with the PFT group (increased by 223%) showing the greatest relative improvement, followed by the PCT (141%) and PIT (63%) training groups, respectively. These findings emphasize the critical role of forearm musculature in the PU performance. As the terminal segment in the kinetic chain, the forearms facilitate force transmission from the latissimus dorsi and biceps to the bar. Insufficient grip strength may thus act as a limiting factor in PU execution [24]. Targeted strengthening of the wrist flexors and extensors likely delayed the onset of grip fatigue, enabling participants to more fully engage the prime movers [24,25]. This finding aligns with prior research demonstrating that dedicated forearm and wrist training can translate to improved sport-specific performance, e.g., increased swing power in baseball players following forearm training [25]. The PCT group also achieved substantial improvements, which may be attributed to enhanced postural control and kinetic efficiency. Improved neuromuscular coordination of the trunk musculature can optimize force transfer and reduce energy dissipation during complex movements [26,27]. In contrast, the PIT group demonstrated modest gains, presumably due to generalized improvements in aerobic capacity and neuromuscular coordination, although part of the improvement may also be attributed to the general PU exercises that all groups performed during the intervention.

Grip strength outcomes further support the specificity of training adaptations. Right-hand grip strength increased significantly only in the PFT group, confirming the effectiveness of direct forearm conditioning in enhancing maximal grip force and mechanical efficiency [24]. PCT did not yield significant improvements in right-hand strength, whereas left-hand strength increased in both the PIT and PFT groups, with the PFT group showing the greatest improvement. These results highlight the principle of specificity and suggest that bilateral neural adaptations may occur even when both limbs are trained concurrently [28].

Grip endurance, assessed via the dead hang exercise or test duration, improved significantly in all groups, indicating that both forearm- and core-focused exercises effectively enhanced the muscular endurance required to sustain bar contact during prolonged efforts. However, there were no statistically significant differences between the groups.

The rapid performance gains observed over the eight-week period are most plausibly explained by neural adaptations rather than structural changes. Early strength improvements are typically driven by enhanced neural efficiency, including increased motor unit recruitment, firing frequency, and intramuscular coordination [15,29]. These neural mechanisms likely contributed to improved activation of the latissimus dorsi, biceps brachii, and forearm muscles. Simultaneously, core training may have bolstered postural stability, reducing energy leakage (i.e., loss of force or mechanical efficiency due to insufficient trunk stability) and facilitating more effective force transmission through the trunk [26,30]. By reducing grip fatigue, a common limiting factor in PU performance, the forearm training enabled participants to more effectively utilize the primary pulling musculature [24].

This study is subject to several limitations that warrant consideration. First, the sample consisted exclusively of thirty physically inactive male university students, which restricts the external validity and generalizability of the findings to broader populations, including trained individuals or female cohorts. This is because sex-specific hormonal and neuromuscular differences, as well as training-status–related variations in adaptation rates, influence strength and pull-up performance outcomes. In addition, minor baseline differences between groups, particularly in pull-up performance, are expected in small samples with wide initial variability and likely reflect natural inter-individual differences rather than a systematic imbalance.

Second, the intervention period was limited to eight weeks, which primarily captured neural rather than structural adaptations. Furthermore, the study did not incorporate neuromuscular assessments such as electromyographic analysis or kinematic profiling, which could have provided deeper insight into the underlying mechanisms of performance enhancement [31]. Participant adherence to dietary and sleep recommendations was monitored via self-reported logs, introducing potential bias and variability due to subjective reporting.

## 5. Conclusions

This study provides compelling evidence that an eight-week intervention integrating PU training with forearm-specific, core-specific, or interval-based exercises significantly enhances upper-body performance metrics, including PU repetitions, grip strength, and grip endurance in physically inactive male participants. Among the three modalities, forearm-specific training yielded the most substantial improvements, underscoring the critical role of grip endurance and forearm musculature in PU execution.

These findings reinforce the principle of exercise specificity in the development of targeted training programs. For individuals seeking to optimize PU performance, the inclusion of forearm-focused exercises may offer distinct advantages by mitigating grip fatigue and enabling full activation of the prime movers [32]. While core and interval training did not produce equivalent gains in grip-related outcomes, they remain integral to a holistic training approach, contributing to postural control, neuromuscular coordination, and general conditioning.

Future research should explore the long-term effects of these interventions across diverse populations and training backgrounds and incorporate neuromuscular assessments to elucidate underlying mechanisms. Such investigations will further inform evidence-based programming for upper-body strength development and sport-specific performance enhancement.

## 6. Practical Implications

From a practical perspective, incorporating short, targeted forearm-specific exercises into pull-up training sessions may substantially enhance performance outcomes by delaying grip fatigue and enabling more effective activation of the prime movers. Coaches and strength practitioners are therefore encouraged to integrate 10–15 min of forearm conditioning twice per week to optimize upper-body pulling performance, particularly in novice or recreational populations.

## Figures and Tables

**Table 1:** Group-specific training parameters of the interval, forearm, and core protocols. (PIT = pull-up + interval training; PFT = pull-up + forearm training; PCT = pull-up + core training.)

PIT	PFT	PCT
High-intensity running (5 sets × 1 min)	Wrist curls (3 × 12)	V-Up (3 × 15)
rest (3 min)	Reverse wrist curls (3 × 12)	Leg raises (3 × 12)
	Reverse grip barbell curls (3 × 12)	Cat and cow (3 × 30)
	Farmer walk (3 × 30 s)	Plank (2 × 1 min)

**Table 2:** Baseline anthropometric characteristics and pre-test values of participants in each group. (PIT = pull-up + interval training; PFT = pull-up + forearm training; PCT = pull-up + core training.)

Variable	PIT (*n* = 10)	PFT (*n* = 10)	PCT (*n* = 10)	*p*-Value
Age	20.2 ± 1.3	20.5 ± 1.0	20.1 ± 1.2	0.5
Height (cm)	180.2 ± 6.9	178.5 ± 10.5	175.7 ± 9.6	0.6
Body mass (kg)	75.5 ± 8.5	75.5 ± 8.4	72.1 ± 9.0	0.1
Pull-up pre-test	4.3 ± 3.2	4.0 ± 2.5	2.7 ± 2.9	0.4

## Data Availability

In accordance with participant consent and privacy protection regulations, the raw data cannot be made publicly available. However, anonymized data can be shared upon a reasonable request from the corresponding author.
